# Step-Wise Deposition Process for Dielectrophoretic Formation of Conductive 50-Micron-Long Carbon Nanotube Bridges

**DOI:** 10.3390/mi11040371

**Published:** 2020-04-01

**Authors:** Tuo Zhou, Ethan Kropp, Jingyuan Chen, Lawrence Kulinsky

**Affiliations:** 1 Department of Mechanical and Aerospace Engineering, University of California, Irvine, 5200 Engineering Hall, Irvine, CA 92627-2700, USA; 2 Department of Materials Science and Engineering, Harbin Institute of Technology, Shenzhen, HIT Campus G908, Shenzhen, Guangdong 518055, China

**Keywords:** carbon nanotube (CNT), dielectrophoresis, electroosmosis, micromanufacturing

## Abstract

Carbon Nanotube (CNT) agglomerates can be aligned along field lines between adjacent electrodes to form conductive bridges. This study discusses the step-wise process of dielectrophoretic deposition of CNTs to form conducting bridges between adjacent electrodes. For the first time, the creation of conductive CNT bridges spanning lengths over 50 microns is demonstrated. The CNT bridges are permanently secured using electrodeposition of the conducting polymer polypyrrole. Morphologies of the CNT bridges formed within a frequency range of 1 kHz and 10 MHz are explored and explained as a consequence of interplay between dielectrophoretic and electroosmotic forces. Postdeposition heat treatment increases the conductivity of CNT bridges, likely due to solvent evaporation and resulting surface tension inducing better contact between CNTs.

## 1. Introduction

Carbon nanotubes (CNTs), high-aspect ratio tubular carbon structures [1], have drawn considerable interest due to their extraordinary physical, mechanical, and electrical properties [2]. CNTs are often used for the enhanced performance of electronic devices such as chemical and biological sensors [3], field-effect transistors [4,5,6], energy storage systems [7], computing devices [8,9], and conductive interconnects [10,11]. Therefore, the integration of CNTs onto predetermined positions in micro- and nanosystems is a critical technology that has been extensively studied [12,13,14,15]. A variety of techniques have been developed to assemble or manipulate individual CNTs or CNT bundles onto desired electrode locations. For example, an atomic force microscope (AFM) tip was used to handle the conveyance of a single multiwalled carbon nanotube to a specific location [16], the capture of nanotube bundles was carried out by magnetic field [17], a combination of conventional and optically induced dielectrophoresis was used to create a line of CNTs between two electrodes [18], CNT transfer technology was developed to transfer CNT bundles [19], CNTs were collected in microgrooves via fluidic assembly [20], and photosensitive chemically binding agents were used to secure CNTs to specific sites [21]. However, the majority of these techniques are slow, laborious, and expensive, and in many cases the aligned CNTs do not produce conductive bridges. In the present work, we describe a step-wise process that can produce conductive bridges, selfassembled out of a CNT suspension and constructed within the high field areas between adjacent electrodes under the influence of dielectrophoresis (DEP).

Dielectrophoresis refers to the force exerted by an external electric field on the induced dipole moment of a particle (i.e., a nanotube) suspended in a dielectric medium [22]. The polarizability of the particle depends on its geometry and on the difference in the complex permittivities between itself and the surrounding medium. Under an applied AC field, these complex permittivities vary throughout a range of frequencies, and determine the direction and magnitude of the DEP force. The magnitude of the induced dipole moment of the CNT is larger along the tube’s axis than in the perpendicular direction, and consequently a rotating moment is created to align the CNT along the electrical field lines [23]. Equation (1) can be used to calculate the DEP force exerted on the polarized nanotube suspended in a medium [24]

(1) $$F_{DEP}=\frac{\pi r^{2}l}{6}\varepsilon_{m}Re\left[f_{m}\right]\nabla E_{rms}^{2},$$

where l and *r* are the length and the molecular radius of the single CNT; $\varepsilon_{m}$ is the permittivity of the medium; $E_{rms}$ represents the root mean square of the applied electric field; and $Re\left[f_{m}\right]$ is the real part of the Clausius- Mossotti factor, $f_{m}$, given by Equation (2)

(2) $$f_{m}=\frac{\varepsilon_{n}^{*}-\varepsilon_{m}^{*}}{\left(\varepsilon_{n}^{*}-\varepsilon_{m}^{*}\right)A_{L}+\varepsilon_{m}^{*}},$$

where $A_{L}$ is the depolarization factor, and $\varepsilon_{n}^{*}$ and $\varepsilon_{m}^{*}$ are the complex permittivities of nanotube and medium, respectively, as defined by Equation (3) below:

(3) $$\varepsilon^{*}=\varepsilon -j\frac{\sigma }{\omega },$$

where ε and σ are the permittivity and conductivity, respectively, and ω is the frequency of the applied electric field. 

In general, if the real part of the complex permittivity of the particle is greater than that of the fluid medium, the DEP force will be “positive” or directed towards the point of the highest field’s intensity. If the real part of the complex permittivity of the particle is smaller than the fluid medium, the particle will have a “negative” DEP force (nDEP) and be forced away from the regions of high field intensity. The sign of the real part of the Clausius–Mossotti factor determines if the particle will experience a positive or negative DEP force.

As the distance between adjacent electrodes increases, so does the difficulty in creating conductive bridges made of nanoparticulates, as the bridges tend to branch into dendritic structures away from the electrodes, as observed previously [25]. Many researchers have bridged gaps reaching submicrons to several micron meter distances, sizes comparable to the lengths of the CNTs [26,27,28,29,30]. Researchers have also created dense forests of nanotube bridges between electrodes that were 25 microns apart [23]. Here, we report for the first time on the process of creating conductive CNT bridges spanning over 50 microns. In order to achieve this result, we employ the technique of step-wise DEP deposition described below.

## 2. Materials and Methods 

### 2.1. Fabrication of IDEAs 

**Figure 1:**
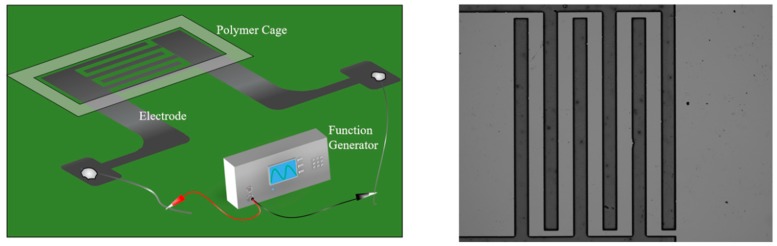
Experimental Setup (**left**) and Interdigitated Array of carbon electrode fingers 120 micron wide with 120 microns inter-electrode spacing (**right**).

The carbon interdigitated electrode arrays (IDEA) (utilized to generate the nonuniform electric field and form the conductive CNT bridges via DEP attraction and alignment) were fabricated with the standard lithographic process using photosensitive SU-8 resin [26], followed by pyrolysis in a nitrogen environment to convert the photo-patterned resist precursor into glassy carbon electrodes [31]. Each IDEA consisted of three electrode finger pairs, as seen in Figure 1. The width and length of each individual electrode finger was 120 μm and 1200 μm, respectively. The distance between the adjacent parallel electrode fingers was 120 μm, and the smallest distance between the electrodes (at the tip of the electrode finger) was 50 microns. 

SU-8 2025 photoresist (Microchem Corp. Ltd, Westborough, MA, USA) was spin coated onto a 4″ silicon wafer covered with 1 μm thick thermal oxide layer (University Wafer, South Boston, MA, USA) using a Laurell photoresist spinner (Laurell Technologies, North Wales, PA, USA) at an initial speed of 500 rpm for 10 s, followed by 4000 rpm spin for 30 s. The soft-baking was carried out on a programmable hot plate (Dataplate, Pmc 732 Series, Dubuque, IA, USA) at 95 °C for 5 min. The wafer was then exposed through a photomask (CadArt, Bandon, OR, USA) to UV light at an energy intensity of 10 mW/cm^2^ for 6s using the Karl Suss MA56 Mask Aligner (Karl Suss, Garching, Germany). The subsequent postbake procedure was performed on the same hot plate by initially heating the wafer at 65 °C for 1 min, followed by a 95 °C step for 5 min. The uncrossed-linked resist was washed away in SU-8 developer (Microchem Corp. Ltd, USA). The remaining cross-linked resist layer was then hot-baked at 150°C for 20 min. The carbonization of the IDEAs was performed by gradually heating up the wafer containing them to 900 °C inside a pyrolysis furnace (Thermo Fisher Scientific, Waltham, MA, USA), within a nitrogen environment. The pyrolysis started at 25 °C for two h, followed by a 69 min ramp to 300 °C, where the temperature was held for one hour before a subsequent 90 min ramp to 900 °C, where the temperature was held again with a dwell time of one hour before being allowed to naturally cool to room temperature overnight. Individual IDEA chips were diced from the wafer. Indium solder was used to attach wires to the carbon IDEA chips. During the pyrolysis step, the polymer precursor shrinks laterally as the carbon electrodes are formed. Since the top of the electrodes shrinks to a larger degree than its base, the sidewall of the carbon electrode becomes tapered. This taper can be clearly seen in the high-resolution SEM pictures in Figure 6 below.

### 2.2. Preparation of Carbon Nanotube Suspension

The CNT suspension was prepared by dispersing 0.005 g of CNT powder containing a mix of single-wall and multiwall nanotubes (Aldrich Chemistry, St. Louis, MO, USA) into 10-mL of isopropyl alcohol (IPA). The suspension was then centrifuged in an Eppendorf 5702 Centrifuge (Eppendorf AG, Hamburg, Germany) at 3000 rpm for 15 min. A pipette (Labnet, Edison, NJ, USA) was used to collect the supernatant with homogeneously distributed CNTs. 

### 2.3. Preparation of Pyrrole Solution

A mixed solution of 0.1M pyrrole monomers (Aldrich Chemistry, St. Louis, MO, USA) and 0.1 M NaDBS (sodium dodecyl-benzene-sulfonate) (Aldrich Chemistry, USA) was prepared by dissolving 0.693 mL of pyrrole monomers and 3.48 g of NaDBS into 100 mL of deionized (DI) water. The solution was stirred at room temperature for 20 min using a magnetic stirrer (Fisher Scientific, Hampton, NH, USA). 

### 2.4. Deposition of CNT Bridges, Resistance Measurement, and Heat Treatment

Wires soldered to an IDEA chip were connected to the function generator (Stanford Research Systems, Sunnyvale, CA, USA) to apply a constant 5V peak-to-peak (Vpp) voltage at various frequencies (see Figure 1). A polymer cage cut out of double-sided stick tape (3M, St. Paul, MN, USA) was used to contain the suspension of CNTs atop of the IDEAs. The CNT suspension was deposited in a series of 10 μL drops. A microscope glass slide (Thermo Fisher Scientific, Fisherbrand, Waltham, MA, USA) was placed atop of the IDEA chip/polymer cage assembly to decrease the evaporation rate of the solution. The resistance between the fingers was measured with a 3320 Innova multimeter (Innova, Irvine, CA, USA). In order to facilitate solvent evaporation after deposition, the IDEA chips were placed on a hot plate (Fisher Scientific, Hampton, NH, USA) at 200 °C for 20 min. The resistance of the CNT bridges was measured again after the heat treatment. This was done to understand the extent to which the evaporation of the residual solution induces closer contact between the nanotubes. 

### 2.5. Polypyrrole (PPy) Deposition

The perpetual capture of CNT bridges was achieved by micropipetting 10 μL of pyrrole/NaDBS solution (see above) over the finger regions of the IDEAs where the CNT bridges formed, and by applying a DC voltage of 0.9 V for about 90 s (deposition was stopped when PPy reached across the CNT bridge). After PPy deposition, the resistance was measured, and the IDEA chips were placed on the hot plate at 200 °C for 20 min followed by another resistance measurement. An optical microscope (Nikon Eclipse, Minato, Japan) and video editing program (SPOT Basic) were utilized to observe and record resulting CNT bridges on IDEA chips. Scanning Electron Microscope (SEM) images were recorded under low current setting using Magellan 400 XHR SEM (FEI, Hillsboro, OR, USA).

### 2.6. Finite Element Analysis Multiphysics Simulation

**Figure 2:**
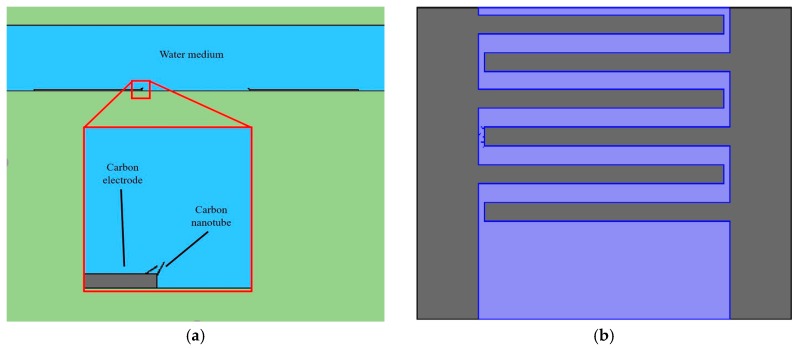
CAD drawings of the interdigitated electrode arrays (IDEA) used in Comsol simulation: side view (**a**) and top view (**b**).

The finite element analysis simulation was carried out with Comsol Multiphysics package (version 5.2) (Comsol, Burlington, MA, USA). 2-D CAD models of the electrode were constructed as presented in Figure 2. The electrode was modelled to be 2.5 μm thick, with the space above the electrodes and top of the water being 75 μm, and the CNTs as being attached to the edge of the electrode. 

The meshing condition was set up to be a physical-controlled mesh with a defined element size set to “extremely fine” and refined near the electrode surface to yield about 18,000 triangular mesh elements. The model was solved by a built-in linear solver. Boundary conditions were set up to be 0 V for the surfaces of three connected electrodes and 4 V for the other three connected electrode fingers. The material of the electrodes was selected to be glassy carbon. In order to simply the model, the permittivity was defined to be 1E8⋅ε_0_, while the liquid medium was isopropyl alcohol with conductivity of 6E6 pS/m and relative permittivity of 18.23⋅ε_0_.

## 3. Results and Discussion

### 3.1. Continuous vs. Step-Wise CNT Bridge Formation

**Figure 3:**
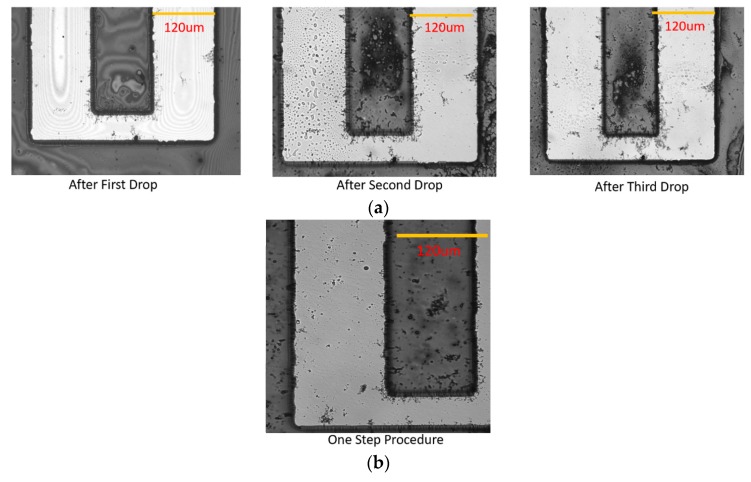
Comparison between the CNT bridges formed by step-wise procedure (**a**) and the CNT bundles attracted by the one-step procedure (**b**). Both deposition procedures used an AC 5 Vpp bias at 100 kHz.

The mechanism leading to the formation of the CNT bridges has been explored. First, a one-step procedure of continuous deposition was tested: when one 30 μL drop of CNT suspension was placed onto the IDEA chip with a 5 Vpp bias and applied frequency of 100 kHz, only short segments of CNT chains formed after several minutes, as seen in Figure 3b. The likely reason for the inability of this single-step continuous deposition process to form a long CNT bridge is the fact that the resulting CNT line that forms via DEP alignment lacks sufficient nanotube-to-nanotube contact. Thus, as the CNT chain is being built, the CNT line becomes more and more resistive until the electric field strength ceases to be sufficient enough for further attraction and alignment of the CNTs.

**Figure 4:**
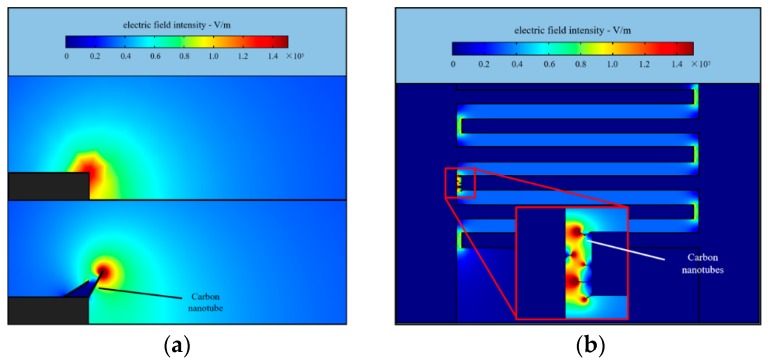
Comsol simulation of electric field intensity around the end of CNTs in the step-wise CNT bridge formation process. (**a**) Field distribution near an individual CNT bundle (**b**) Results of the simulation show that during bridge formation, regions of highest field intensity shift from the edges of electrodes to the ends of the CNT bridges.

We are proposing an alternative to continuous CNT deposition—a step-wise deposition where first one droplet of CNT suspension is deposited over the electrodes to enable CNT segments to begin forming under the influence of DEP forces. We then dry the solution from CNT suspension, or simply wait until the droplet of solvent evaporates (it usually takes just a few minutes as the CNT suspension is not covered by the glass slide). As solvent evaporates, surface tension of shrinking droplets surrounding the deposited CNTs will pull CNTs together, enabling better contact, and consequently forming a CNT spike that results in an area of high field as demonstrated by the results of the Comsol simulation shown in Figure 4.

A step-wise deposition of CNT bridges was successfully performed under a wide range of frequencies. Figure 3a presents the results of CNT bridge formation after the deposition and drying of three 10 μL drops of CNT suspension (in contrast to a single 30 μL CNT suspension drop used in a single step continuous deposition trial). The applied voltage and frequency of 5 Vpp and 100 kHz were also used as in the single-step continuous deposition trial. It can be seen in the sequence of optical micrographs in Figure 3a how the CNT bridge forms gradually after deposition of each CNT droplet, until a continuous conductive CNT bridge is formed that spans the entire 50 μm inter-electrode spacing.

### 3.2. Applied Frequency Influence on the Morphology of CNT Bridges

**Figure 5:**
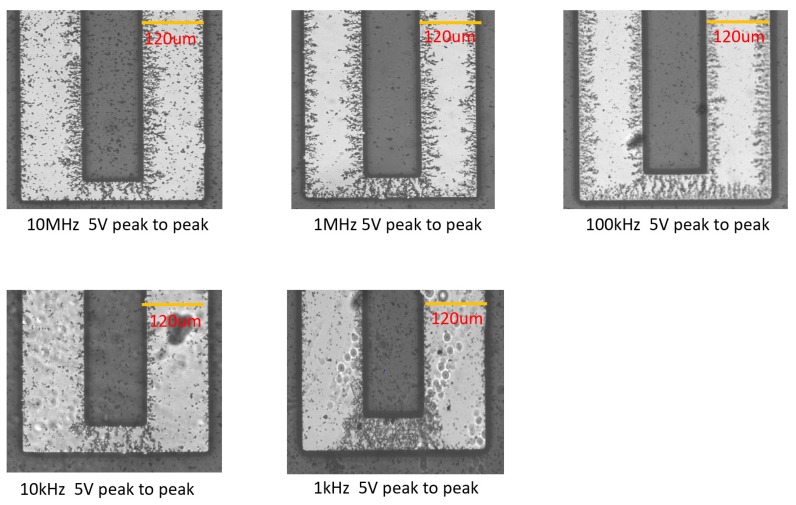
CNT bridges deposited under 5 Vpp AC bias at 1 kHz, 10 kHz, 100 kHz, 1 MHz, and 10 MHz applied frequencies.

**Figure 6:**
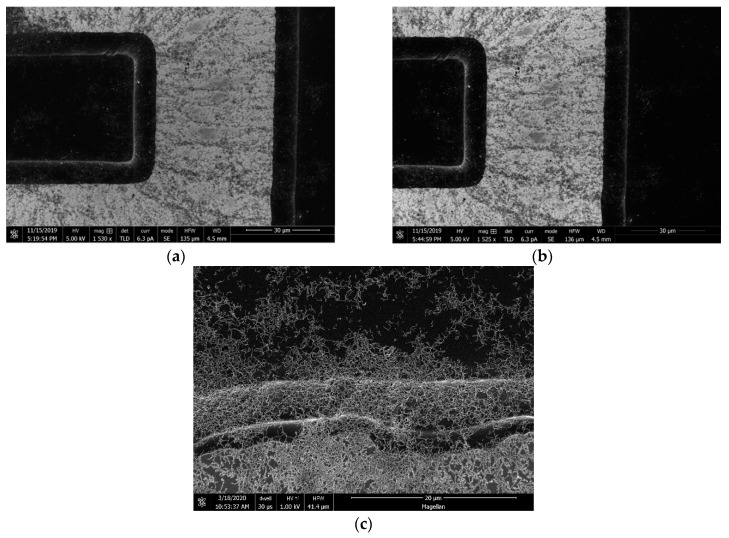
The SEM images of CNT bridges deposited under 100 kHz frequency. (**a**,**b**) SEM images of electrodes at 1530 and 1525 magnification, respectively; (**c**) SEM image of the edge of electrode at 5000 magnification.

The formation of the bridges was demonstrated under the AC bias of 5 Vpp at frequencies of 1 kHz, 10 kHz, 100 kHz, 1 MHz, and 10 MHz (see optical micrographs on Figure 5 and Scanning Electron Microscopy (SEM) images in Figure 6). The morphologies of the CNT bridges depended on the applied frequencies. At relatively low frequencies, such as 10 kHz and 1 kHz, electro-osmotic (EO) forces [32,33,34] generate fluidic circulation near the edges of the fingers of the IDEAs where the electric field intensity is strongest. In the presence of these electro-osmotic forces, the alignment of the CNTs is disturbed. Outside the frequency range of EO flow, and within frequencies ranging from 100 kHz to 10MHz, the positive DEP attraction of CNTs to the edges of the fingers of the electrodes dominates vanishingly small EO forces. Since the magnitude of the electro-osmotic force increases with decreasing frequency [34], larger amounts of CNTs needed to be deposited at the bridge sites for the bridges to form. This relationship between EO and DEP forces at various frequencies helps to explain the observed morphologies of the CNT bridges. 

### 3.3. Influence of Postdeposition Heat Treatment on Resistance of CNT Bridges

**Figure 7:**
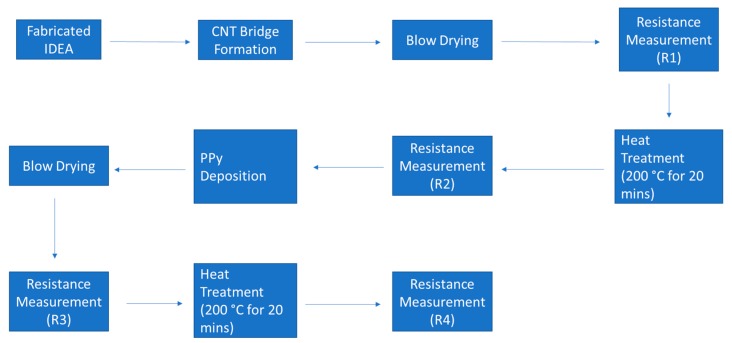
The flow chart of the experimental procedure.

**Figure 8:**
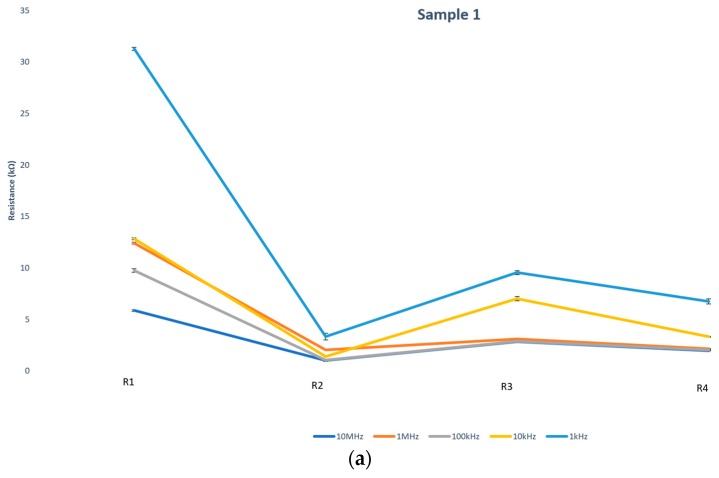

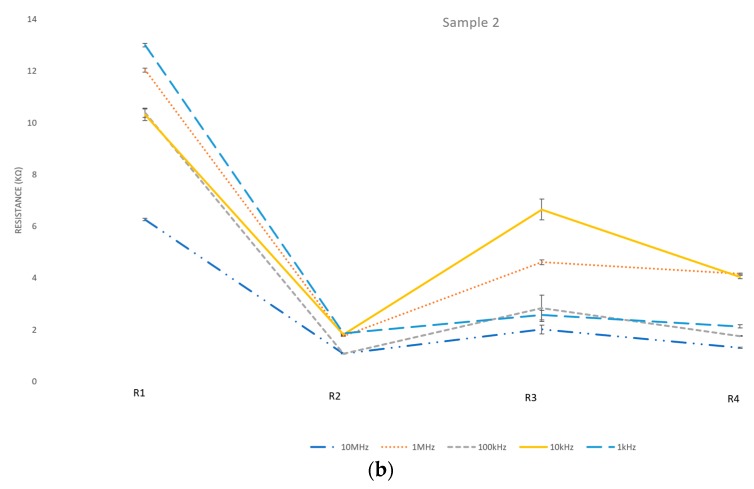
The resistance of CNT bridges of sample 1 (**a**) and sample 2 (**b**) as function of deposition parameter and postdeposition heat treatment including after CNT deposition (R1), drying on hot plate for 20 min at 200 °C (R2), after polypyrrole deposition (R3), and after final drying on hot plate for 20 min at 200 °C (R4).

Dependence of postdeposition heat treatment on the resistance of fabricated CNT bridges has been explored on two samples (identified as Samples 1 and 2 in Table 1 and in the resistance measurement plots in Figure 8). First, initial resistance of the IDEA array was measured. As expected, prior to CNT bridge deposition, the resistance was infinite. After the CNT bridges formed, the IDEA chip was blow dried with a nitrogen gun for about a minute until the chip was visibly dry, then the resistance of the IDEA was measured by placing the probes of the multimeter on the contact pads of the IDEA chip. That measurement is termed R1. The IDEA chip was then placed on a hot plate at 200 °C for 20 min; afterward, the resistance was measured again (R2). In order to permanently secure CNT bridges in place, a layer of polypyrrole was electrodeposited as discussed in the Materials and Methods section above and on Section 3.4 below. After PPy deposition over CNT bridges, the chip was blow dried and the resistance was measured again (R3) and once again (R4) after another heat treatment. The flowchart of these steps is presented in Figure 7. Table 1 contains the resistance measurements for CNT bridges deposited under the variety of applied frequencies, while Figure 8 represents a graph of these measurements for clear identification of the trends.

**Figure 9:**
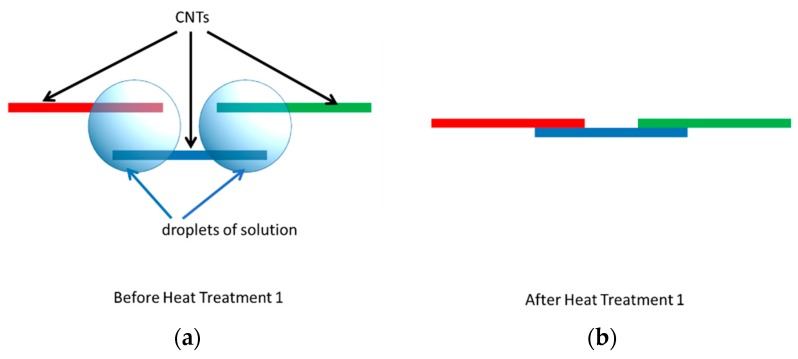
The illustration of the influence of postdeposition heat treatment on CNT bridges. (**a**) Remaining droplets present after blow drying keep CNTs separated; (**b**) Evaporation of remaining solvent after heat treatment and the resulting changes in surface tension act to pull CNTs together, improving CNT-CNT contact.

It is clear that as a result of the heat treatment (200 °C for 20 min), the resistance of the CNT bridges decreases dramatically. For example, for CNT bridges deposited under 5 Vpp at an AC frequency of 1 MHz, the resistance is reduced six-fold from around 12 kΩ prior to heat treatment to roughly 2 kΩ after the heat treatment. Our hypothesis is that this decrease in the resistance can be attributed to the evaporation of any residual CNT solution existing between the individual CNTs. These pockets of liquid remaining after blow drying steps start to further shrink and evaporate under the heat treatment. As the liquid surrounding the CNTs evaporates, the surface tension of these shrinking droplets pulls CNTs together as depicted in Figure 9, leading to a dramatic increase in conductivity of CNT bridges. This formation of microaggregates due to surface tension during solvent evaporation was observed by other researchers [35].

### 3.4. PPy Deposition over CNT Bridges

**Figure 10:**
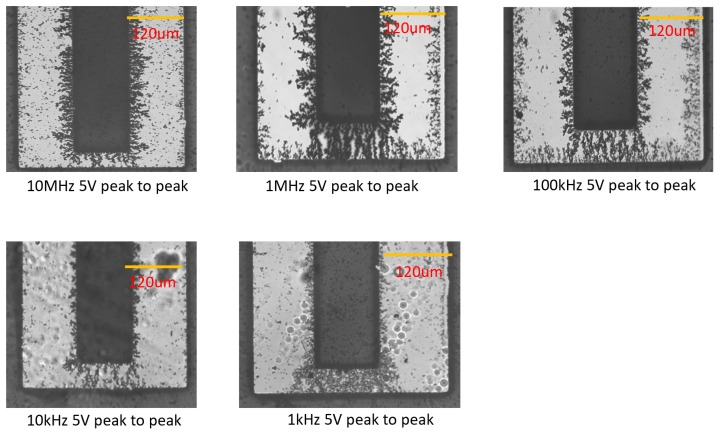
The CNT bridges after electrodeposition of PPy.

**Figure 11:**
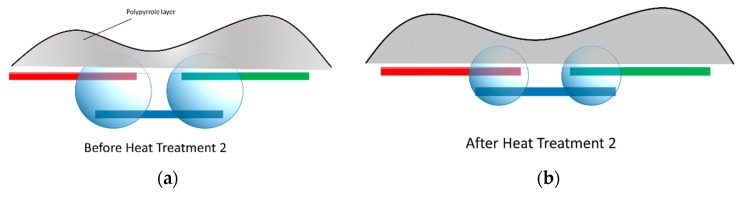
The illustration of the influence of heat treatment on CNT bridges after PPy deposition. (**a**) PPy deposition envelops CNT bridges with remaining solvent after blow drying (**b**) Less dramatic changes in resistance were observed before and after the final heat treatment. This is attributed to the PPy layer which prevents complete evaporation of solvent.

After CNT bridges were deposited under the influence of electrokinetic forces as discussed in previous sections, pyrrole solution was dispensed over the IDEA and electropolymerization was performed to permanently entrap and fix the fabricated CNT bridges [36]. The PPy was initially electropolymerized at the surface of the electrode fingers and subsequently grew along the CNT bridges until a PPy layer completely covered the CNT bridge, as shown in Figure 10. The resistances were measured after the PPy deposition (R3) and after the 20 min heat treatment at 200 °C (R4). These electrical resistance measurements are listed in Table 1 and graphed on the plot in Figure 8. The increase in the resistance (R3 > R2) after the PPy deposition can be attributed to the introduction of PPy solution that tends to separate CNTs within the bridge. The heat treatment after PPy deposition was not as effective as before (R4 > R2) because there was a polymer film covering CNT bridges that slowed down solvent evaporation, as illustrated in Figure 11. 

## 4. Conclusions

The present study demonstrates a novel step-wise process that incorporates electrokinetic phenomena to deposit and form long chains of CNTs along the electrical field lines of an external AC field. The deposition-drying-deposition sequence of steps allows for the creation of conductive CNT bridges between adjacent electrodes. In this work, for the first time, we demonstrate the creation of conductive CNT bridges over 50 microns in length. It has been observed that the morphologies of CNT bridges deposited at frequencies of 100 kHz and above are more orderly than for CNT bridges deposited at lower frequencies. This difference in the morphologies is attributed to the disruptive circulation of fluid caused by electroosmosis at lower frequencies. The role of postdeposition heat treatment was also explored. The observed increase in the conductivity of CNT bridges after the heat treatment step of 200 °C for 20 min is consistent with the hypothesis that the evaporating solvent and resulting surface tension pulls together CNTs and consequently decreases CNT–CNT contact resistance. Finally, electrodeposition of polypyrrole was performed to permanently secure CNT bridges. We believe that the described step-wise electrokinetic deposition process, capable of producing long chains of conductive CNT bridges, will find applications in micro- and nanoelectronics, sensors, and energy storage and conversion. 

## Figures and Tables

**Table 1:** Resistivities of samples 1 and 2 showing mean values and standard deviation after CNT deposition (CNT), drying on hot plate for 20 min at 200 °C (CNT + HT), polypyrrole deposition (CNT + HT + PPy), and final drying on hot plate for 20 min at 200 °C (CNT + HT + PPy + HT).

	CNT (kΩ)	CNT + HT (kΩ)	CNT + HT + PPy (kΩ)	CNT + HT + PPy + HT (kΩ)
Sample 1				
10 MHz	5.86 ± 0.09	1.03 ± 0.11	2.82 ± 0.02	1.98 ± 0.01
1 MHz	12.43 ± 0.1	2.04 ± 0.01	3.11 ± 0.04	2.17 ± 0.10
100 kHz	9.75 ± 0.12	1.05 ± 0.01	2.88 ± 0.04	2.05 ± 0.02
10 kHz	12.83 ± 0.08	1.41 ± 0.02	7.01 ± 0.21	3.31 ± 0.05
1 kHz	31.27 ± 0.15	3.34 ± 0.33	9.54 ± 0.20	6.74 ± 0.25
Sample 2				
10 MHz	6.26 ± 0.05	1.08 ± 0.01	2.01 ± 0.17	1.31 ± 0.02
1 MHz	12.04 ± 0.08	1.76 ± 0.02	4.61 ± 0.10	4.17 ± 0.03
100 kHz	10.39 ± 0.18	1.07 ± 0.01	2.83 ± 0.51	1.76 ± 0.02
10 kHz	10.31 ± 0.23	1.82 ± 0.02	6.65 ± 0.40	4.04 ± 0.06
1 kHz	13 ± 0.07	1.88 ± 0.01	2.57 ± 0.19	2.13 ± 0.06
